# On the Comparative Effects of Light and Heat

**Published:** 1803-01-01

**Authors:** 


*On the comparative Effects of Light and Heat, by* Cit. BERTHOLLET; *communicated by our Correspondent at Paris.*

COUNT Rumford has observed, that the solution of gold mixed with different white bodies, becomes purple on being exposed to the solar light, or to the heat of a candle, although it undergoes no such change in the dark; he has remarked analogous changes in the solution of silver. These changes, as well as the transition of white to black, which the muriat of silver undergoes when placed under water, and exposed to light, had been ascribed to the disengagement of oxygen from the oxyd of gold or of silver, and to the two metals approaching to a metallic state. Cit. Berthollet, however, never perceived that oxygen was disengaged in this case; the water became acid, but contained nothing but simple muriatic acid, and not oxygenated muriatic acid. From this and similar observations, he concludes that the change of colour in the muriats of gold and of silver, is owing to the disengagement of a part of muriatic acid, which seems to be assisted by the presence of water. Cit. Berthollet thinks, that the acid combined with the oxyd of silver, by its affinity with that oxyd, prevents the gold and silver from recovering their metallic state, in the same manner as terreous and vitrifiable substances prevent the reduction of metallic oxyds. In a strong heat, however, these auxiliar affinities, as Berthollet expresses himself, are not sufficiently powerful, and hence we may understand, why colours on porcelain, which originate from the oxyd of gold, are more fugitive than those of other oxyds, and cannot endure the operations that require a great fire. He mentions, afterwards, the experiments of Count Rumford, according to which he has reduced the oxyds in the solutions of gold and silver, brought in contact with coal, and exposed to the action of solar light, or to the heat of boiling water. These observations seem to confirm the identity of the substance of light with that of heat, or at least the identity of their effects. Cit. Berthollet however adds, that we may discover in the circumstances attending the action of light, the reason of the different effects, as the latter disengages the oxygen of the oxygenated muriatic acid, and of the nitric acid, while heat only suffers them to pass over in the distillation, without decomposing them.

